# The Effects of Multiple Freeze–Thaw Cycles on the Biomechanical Properties of the Human Bone-Patellar Tendon-Bone Allograft

**DOI:** 10.1002/jor.21373

**Published:** 2011-03-04

**Authors:** Ho-Joong Jung, Gautum Vangipuram, Matthew B Fisher, Guoguang Yang, Shanling Hsu, John Bianchi, Chad Ronholdt, Savio L-Y Woo

**Affiliations:** 1 Department of Bioengineering, Swanson School of Engineering, Musculoskeletal Research Center, University of PittsburghPittsburgh, Pennsylvania; 2 Department of Orthopaedic Surgery, College of Medicine, Chung-Ang UniversitySeoul, Korea; 3 Revivicor Inc.Blacksburg, Virginia; 4 LABS Inc.Centennial, Colorado

**Keywords:** anterior cruciate ligament reconstruction, bone-patellar tendon-bone allograft, multiple freeze–thaw cycles, biomechanics

## Abstract

**Introduction:** Soft tissue allografts, such as the bone-patellar tendon-bone (BPTB) graft, have been frequently used for anterior cruciate ligament (ACL) reconstruction. As allografts are subjected to freezing and thawing for multiple cycles, the objective of this study was to measure the changes of the biomechanical properties of the human BPTB allograft after 4 and 8 freeze–thaw cycles in comparison to a single freeze–thaw cycle. **Methods:** Three BPTB specimens were procured from 21 human donors and divided into three groups: 1, 4, or 8 freeze–thaw cycles. Each freeze–thaw cycle consisted of freezing at −20 ± 10°C for more than 6 h and thawing at 22 ± 3°C for at least 6 h. Tensile testing of the BPTB specimens consisted of loading between 50 N and 250 N for 100 cycles and then loading to failure. **Results:** Cyclic loading revealed a similar amount of creep (∼0.5 mm) among the three freeze–thaw cycles groups (*p* = 0.38). The stiffness of the BPTB graft for the 1, 4, and 8 freeze–thaw cycle groups were 244 ± 42 N/mm, 235 ± 39 N/mm, and 231 ± 40 N/mm, respectively (*p* = 0.43). Similar findings were obtained for the ultimate load of the BPTB graft (*p* = 0.14) and the tangent modulus of the PT substance (*p* = 0.41). **Discussion:** The results of this study suggest that there would be little measurable effect on the structural properties of the BPTB graft or mechanical properties of the PT tissue substance following 8 freeze–thaw cycles. These BPTB allografts could potentially be re-frozen without a loss in their biomechanical properties, given appropriate storage and care. © 2011 Orthopaedic Research Society Published by Wiley Periodicals, Inc. J Orthop Res 29: 1193–1198, 2011

Soft tissue allografts are commonly used for anterior cruciate ligament (ACL) reconstruction procedures, and their demand has increased in recent years.^1^–^4^ Amongst the allograft choices, the bone-patellar tendon-bone (BPTB) complex is preferred because it possesses bone blocks at both ends for better initial fixation as well as faster bone-to-bone healing.^5^,^6^

Prior to implantation in a patient, the BPTB allografts can be subjected to multiple freeze–thaw cycles. Following recovery of the donor tissue (e.g., femur, tibia, knee-en-bloc), the tissue is wrapped, frozen, and shipped to a processing center, where it is often thawed for inventory and re-frozen. After, 1 or 2 processing steps occur, in which the tissue is thawed and processed into several allograft tissues for implantation (e.g., femoral rings, BPTB grafts, etc.), and again refrozen. Additional freeze–thaw cycles may be necessary for quality control requirements, decontamination steps, packaging, and sterilization prior to distribution or may occur at the hospital in cases of cancelled surgery or improper sizing, among other clinical reasons. As a result, the Scientific and Technical Affairs Committee (STAC) of the American Association of Tissue Banks (AATB), which represents the major tissue processing agencies in the United States, has affirmed that a majority of musculoskeletal allograft tissues, such as the BPTB, may undergo as many as 4 freeze–thaw cycles before its use.

The literature has reported differing results regarding the effects of freezing on the biomechanical properties of ligaments and tendons. Some studies showed little or no adverse effects on the mechanical properties,^7^–^13^ while other studies suggested that some negative changes to these properties could occur.^14^–^19^ Studies from our research center reported no difference in the biomechanical properties of the medial collateral ligament in a rabbit model between fresh, unfrozen samples and those that underwent 1 or 2 freeze–thaw cycles.^10^,^11^ Other studies have shown that even though the mechanical properties of the frozen rat patellar tendon (PT) were not statistically significant compared to those tested before freezing, ice crystals formed that led to wider interfibrillar spaces and altered fiber alignment that would not return after thawing.^9^

However, it is less clear if multiple freeze–thaw cycles, such as those experienced by allografts, would have an impact on their biomechanical properties. Such information could help to guide clinicians as well as tissue processors as to any detrimental effects of multiple freeze thaws. To address this, the objective of this investigation to measure the biomechanical properties of the unprocessed human BPTB allograft after 4 and 8 freeze–thaw cycles and compare them with those subjected to a single freeze–thaw cycle.

## MATERIALS AND METHODS

Human cadaveric knee specimens consisting of the whole patella, PT, and the tibial bone (with a length of at least 10 cm from the joint line) were obtained from 21 donors (13 males, 8 females) via tissue establishments that are accredited by the AATB. The mean age of the donors was 63 ± 14 years (range: 27 to 79 years). Only specimens which had been previously frozen once after recovery were utilized in this study. Specimens were not subjected to any other processing steps, such as chemical solutions and antibiotics or terminal sterilization. All donors were confirmed to have provided consent for research by the procurement agencies or tissue processing centers.

**Figure 1:**
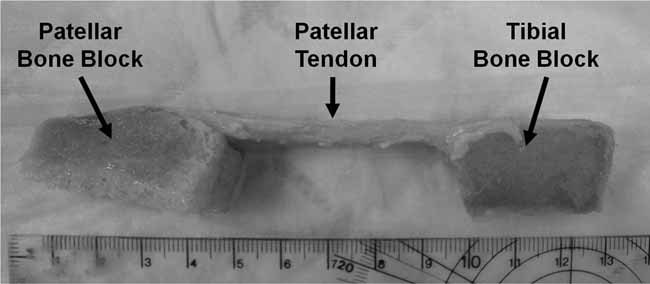
Typical BPTB specimen following isolation.

Upon thawing, the PT was first checked to ensure that there were no defects, such as tears in the ligament or cracks in the patella or tibial bone block. Test specimens used did not have any noticeable defects. Then, the width of the PT in the medial-lateral direction was measured, and a 10 mm wide portion was excised, along with the corresponding patellar and tibial bone blocks. If the width of the PT exceeded 24 mm, the tendon was divided into two hemi-sections. At least three BPTB specimens were obtained from each donor. The bone blocks were trimmed to a similar size as the initial graft dimensions of allografts used in surgery, and were at least 12 mm wide × 12 mm thick × 20 mm in length (Fig. 1).

The BPTB specimens were block randomized to the 1, 4, or 8 freeze–thaw cycle groups, such that an equal amount of specimens were placed in each group and one specimen per donor was in each group. The number of cycles were chosen to simulate a fresh-frozen tissue (1 freeze–thaw cycle group), the typical number of freeze–thaw cycles experienced during allograft processing (4 freeze–thaw cycle group), and twice the typical standard (8 freeze–thaw cycle group). A freeze–thaw cycle was defined as placing the specimen in a freezer at −20 ± 10°C for more than 6 h, and thawing at 22 ± 3°C for at least 6 h.

After a sample had completed its assigned number of freeze–thaw cycles, orthodontic resin (Lang Dental, Wheeling, IL) was used to fix the sample within a 2 × 1 × 1 cm custom made jig. The fixed sample was wrapped in saline-soaked gauze and allowed to sit in a refrigerator (4°C) overnight to ensure the resin would cure fully.

**Figure 2:**
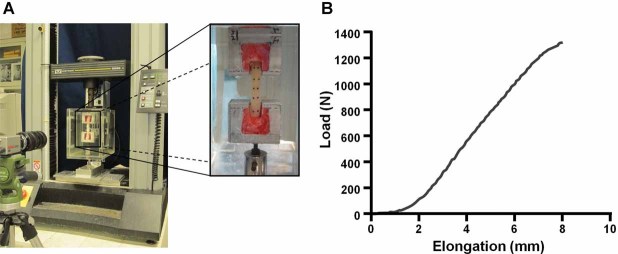
(A) Experimental apparatus for tensile testing. The patellar and tibial bone blocks were gripped using custom clamps. Markers were placed on PT substance for strain tracking. (B) Typical load-elongation behavior of a BPTB specimen.

Prior to tensile testing, measurements of tendon length between the bone blocks, width, and thickness were done using digital calipers. A laser micrometer was used to determine the cross-sectional area (CSA) of the tendon.^20^,^21^ Three readings were taken at the proximal (patellar), middle, and distal (tibial) portions of the tendon, and an average of these readings was used as the CSA for stress measurements. Ten reflective markers were evenly placed on the surface of the tendon and insertion sites in two rows of five markers for strain tracking via a digital motion analysis system (DMAS Motion Analysis, Spicatek, Inc., Maui, Hawaii; Fig. 2A).

The specimens were then mounted on a uniaxial materials testing machine (Model 5565; Instron, Canton, MA) within a 0.9% saline bath held at 37°C (Fig. 2A).^22^ A small load was applied to facilitate alignment of the tendon along the direction of loading. Then, the specimen was unloaded and allowed to equilibrate to the environment for 30 min.

After equilibration, a 2 N preload was applied, and the gauge length was reset. Specimens were pretensioned at 89 N, while measuring the load. Tension was then adjusted back to 89 N after 5 min and 15 min. After a total of 25 min, the specimen was unloaded to 0 N for 30 min before commencing cyclic loading. This portion of the protocol was meant to simulate the forces applied by the surgeon before graft implantation in ACL reconstruction procedures.^23^ For the cyclic loading test, the specimen was cycled between 50 and 250 N for 100 cycles at an elongation rate of 20 mm/min. This was meant to simulate forces encountered during graft implantation and early rehabilitation.^23^ The number of cycles was based on preliminary studies, which compared the amount of tissue elongation up to 1000 cycles and found that the vast majority of tissue elongation occurred within the first 100 cycles. Creep was calculated as the difference in elongation at 250 N between the 3rd and 100th cycles.^23^ The specimen was allowed to recover for 30 min before loading to failure at an elongation rate of 50 mm/min. The failure mode was recorded for each specimen.

Parameters representing the structural properties of the BPTB complex were obtained from the resulting load-elongation curve. Stiffness was defined as the maximum slope over a 2 mm interval on the load-elongation curve. Ultimate load was defined as the maximum load before failure. Parameters representing the mechanical properties of the PT tissue were obtained from the stress-strain curve. Stress was calculated by dividing the load by the CSA of the PT. Strain was calculated as the distance between the midsubstance markers divided by their initial distance. The tangent modulus was calculated as the maximum slope of the stress-strain over a 1% interval. There was an experimental error with respect to strain measurements for one specimen, and the data for tangent modulus for that specimen were excluded from further analysis.

An a-priori power analysis performed using G*Power software indicated that 21 specimens per group would be needed to detect an effect size of 0.8 with an overall *α* = 0.05 and a power of 0.8.^24^ Statistical analyses of tissue dimensions, creep, ultimate load, stiffness, and tangent modulus were conducted using a repeated measures ANOVA in SPSS (SPSS software, Version 14.0, SPSS, Inc., Chicago, IL) followed by a Bonferroni post-hoc test, if warranted. To assess differences in failure mode, a Pearson chi-square test was used. A statistically significant result was indicated by *p* < 0.05.

## RESULTS

Through gross observation, there were no changes to the appearance of the PT tissue even after an increased number of freeze–thaw cycles. The dimensions of the PT tissues for each group are detailed in Table 1. There were no statistical differences in tissue length between the bone blocks, width, nor thickness among the three freeze–thaw cycle groups (*p* = 0.31, *p* = 0.74, and *p* = 0.63, respectively). Furthermore, the CSA of the PT tissue for the 1, 4, and 8 freeze–thaw cycle groups also differed by<7% (50–53 mm^2^), with no statistical differences among them (*p* = 0.12).

**Table 1:** Gross Measurements of the PT Substance for the 1, 4, and 8 Freeze–Thaw Cycle Groups (Mean ± SD)

	Number of freeze–thaw cycles		
	
	1	4	8
Length between bone blocks (mm)	39.2 ± 7.7	37.8 ± 6.4	40.1 ± 7.0
Width (mm)	10.2 ± 0.7	10.3 ± 0.4	10.1 ± 0.3
Thickness (mm)	3.4 ± 0.7	3.4 ± 0.6	3.5 ± 0.5
Cross-sectional area (mm^2^)	49.9 ± 8.5	53.5 ± 7.5	52.1 ± 6.5

From tensile testing, the data on creep, stiffness, ultimate load, and failure mode of the BPTB graft as well as the tangent modulus of the PT tissue are detailed in Table 2. For the cyclic loading test, there was a small amount of creep (on the order of 0.5 mm) after 20 cycles of loading and unloading, which occurred for all three freeze–thaw cycle groups. These values represented <2% of the overall tendon length. Additional cyclic loading up to 100 cycles did not increase the amount of creep. Statistical comparisons of the amount of creep among the 1, 4, and 8 freeze–thaw cycle groups showed no significant differences (*p* = 0.38).

**Table 2:** Effects of the Number of Freeze–Thaw Cycles on the Structural Properties of the BPTB Allografts (Mean ± SD)

	Number of freeze–thaw cycles		
	
	1 (*n* = 21)	4 (*n* = 21)	8 (*n* = 21)
(A) Parameters describing the structural properties of the BPTB			
Creep (mm)	0.4 ± 0.2	0.5 ± 0.3	0.6 ± 0.5
Stiffness (N/mm)	244 ± 42	235 ± 39	231 ± 40
Ultimate load (N)	1276 ± 322	1141 ± 319	1170 ± 357
(B) Mode of failure (number of cases)			
Bony avulsion	9 (5 T, 4 P)	6 (4 T, 2 P)	5 (5 T, 0 P)
Detachment at tissue insertion to bone	8 (8 T, 0 P)	9 (9 T, 0 P)	12 (10 T, 2 P)
Substance tear	4	6	4

T, tibial side; P, patellar side.

**Figure 3:**
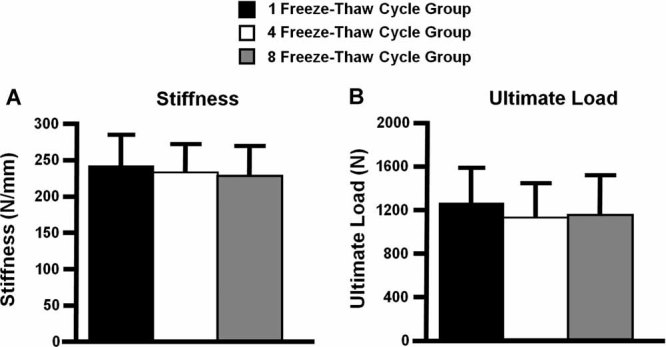
Graphical representation of the stiffness (A) and ultimate load (B) representing the structural properties of the BPTB grafts for the freeze–thaw cycle groups (mean ± SD).

Load-elongation curves of the BPTB grafts revealed a typical non-linear toe region up to approximately 1 mm of elongation, followed by a linear region between 3 to 6 mm (Fig. 2B). With further loading, yielding behavior occurred prior to specimen failure. The parameters obtained included the stiffness and ultimate load, which are used to describe the structural properties of the BPTB graft (Table 2A). For the stiffness, the mean values were 244 N/mm, 235 N/mm, and 231 N/mm for the 1, 4, and 8 freeze–thaw cycle groups, respectively (Fig. 3A), with no significant differences detected (*p* = 0.43). For the ultimate load, the corresponding mean values were 1276 N, 1141 N, and 1170 N (Fig. 3B), and again, no statistically significant differences could be detected among the three freeze–thaw cycle groups (*p* = 0.14).

The modes of failure included tendon substance tear, detachment of the tendon from the bone at the insertion site, and pure avulsion of the bone (Table 2B). The most common failure mode was detachment of the tendon at its insertion to the bone, which occurred in 38%, 43%, and 57% of samples in the 1, 4, and 8 freeze–thaw cycle groups, respectively. Of these, over 90% occurred on the tibial side, with the remainder on the patellar side. The percentages for bony avulsion were 43%, 29%, and 24%, the majority of which occurred on the tibial side (70%). Finally, midsubstance failures were noted in 19%, 29%, and 19% for the 1, 4, and 8 freeze–thaw cycle groups, respectively. Statistical analysis again revealed no significant differences in the failure modes among the three freeze–thaw groups (*p* = 0.60).

**Figure 4:**
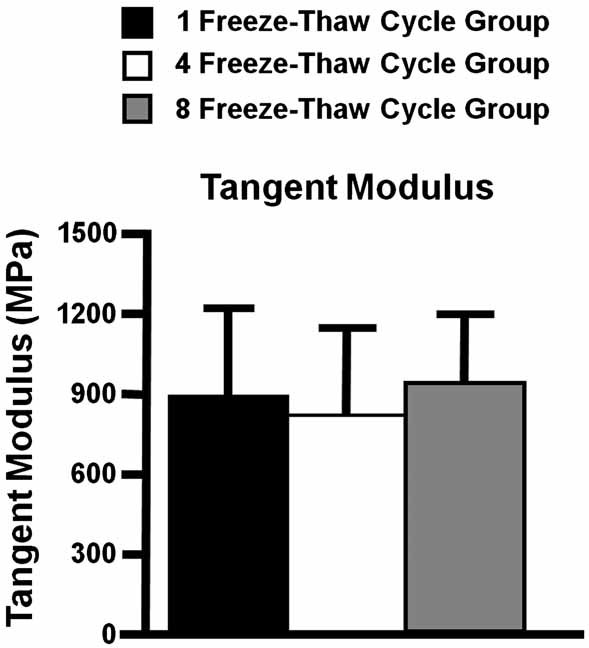
Graphical representation of the tangent modulus of the PT for the freeze–thaw cycle groups (mean ± SD).

In terms of the mechanical properties of the PT substance, a representation of the quality of the tissue, the tangent moduli were 900 ± 330 MPa, 829 ± 325 MPa, and 951 ± 256 MPa for the 1, 4 and 8 freeze–thaw cycle groups, respectively (Fig. 4). Again, there were no statistically significant changes in tangent modulus among the three freeze–thaw cycle groups (*p* = 0.41).

## DISCUSSION

This study was designed to assess the effects of multiple freeze–thaw cycles on the biomechanical properties of the human BPTB graft. The data obtained indicate that up to 8 freeze–thaw cycles would not cause any statistically significant changes in creep, stiffness, ultimate load, and mode of failure of the BPTB graft, nor the tangent modulus of the PT substance as compared to 1 or 4 freeze–thaw cycles. The lack of changes in the stiffness of the BPTB graft together with the creep elongation with up to 8 freeze–thaw cycles is of high clinical importance, as these subfailure properties occur at the levels of loading similar to those the graft will experience during activities of daily living.

An important aspect of the current study is that the specimens in each group were obtained from the same set of donors; thus, allowing for paired statistical analyses between groups and eliminating inter-specimen variability due to other factors such as gender, age, and size.^22^,^25^,^26^ Even with such an increase in statistical power, no statistical significance could be detected the biomechanical properties measured regardless of the number of freeze–thaw cycles performed.

Previous studies examining the effects of 1 or 2 freeze–thaw cycles on the biomechanical properties of ligament and tendon tissues have had varied results, with some finding little or no changes^7^–^13^ while others noted substantial adverse effects.^14^–^19^ Many biological and experimental factors could contribute to these discrepancies, including the source of the tissues (human or animal), tissue location and type, methods of freezing and temperature as well as the duration of storage, methods of thawing, capabilities of the mechanical testing system capabilities, protocols used, and methods of stress and strain measurement, among others. This highlights the need to carefully interpret the results of these studies.

In the current study, even with additional freezing steps along with an increase in time at room temperature between freeze–thaw cycles, there was no adverse effect on the biomechanical properties of the unprocessed BPTB grafts. During standard tissue procurement, processing, packaging, and sterilization, BPTB allografts encounter a number of freeze–thaw cycles. Although the amount of time the tissue remains at ambient conditions is variable, the freeze–thaw conditions used in this study represent a worst case scenario. Thus, the data support the concept that a number of freeze–thaw cycles may not adversely affect the biomechanical integrity of unprocessed BPTB allografts, provided that the tissues are carefully handled. These data could also be useful for experimental studies on the biomechanics of soft tissues, particularly when the testing protocol requires that the specimens need to be re-stored multiple times. However, it should again be noted that extreme care in the handling and storage of the test specimen must be carried out in order to retain its tissue quality and biomechanical properties and variations to the freezing and thawing steps could lead to alternative results.

The values for the structural properties of the BPTB graft and mechanical properties of the PT tissue obtained in this study were consistent with those in the literature.^22^,^23^,^25^,^26^ For example, Jones et al.^23^ isolated a BPTB graft in a similar manner as the current study and their data on stiffness (235.3 ± 37.6 N/mm) and ultimate load (1685.7 ± 471.6 N) are very close to the current data. In terms of the tangent modulus of the PT tissue, the values in the current study (829-951 MPa) were slightly higher than those reported in the literature (307–680 MPa).^22^,^25^,^26^ However, it should be noted that differences in experimental procedures between studies, especially in the specimen preparation, methodology for clamping, techniques for strain measurement, and testing protocol, can change the outcome of the measured tangent modulus. Nevertheless, the results of the current study are within reason versus the findings in the literature.

Limitations to the present study include a lack of analysis of the biochemical changes in the PT substance following a number of freeze–thaw cycles. As such, the amount of changes in water, ground substance, and collagen content were not known. Also, we did not investigate the effect of different rates of freezing and thawing as well as lower temperatures (e.g., −80°C), which may produce a different outcome. In the future, these factors should be considered along with age- and gender-related changes. Additionally, it is not known how processing steps used by tissue banks (e.g., washes in chemical solutions and antibiotics, mechanical agitation, and possibly exposure to a terminal sterilization process) may influence the biomechanical properties of BPTBs in combination with multiple freeze–thaw cycles. In future studies, tissue banks can use this study design as a template to test BPTB allografts post-processing. Finally, animal studies are suggested to examine the biologic incorporation of an allograft that has undergone multiple freeze–thaw cycles.

Still, the results of this in-vitro study are encouraging and provide a scientific basis that an unprocessed BPTB allograft could undergo up to 8 freeze–thaw cycles without compromising its biomechanical properties. Thus, the data support the notion that an unprocessed BPTB allograft could undergo multiple freeze–thaw cycles, provided that storage and handling processes are carefully administered, without compromising its biomechanical properties.
